# Evolutionary History of Indian Ocean Nycteribiid Bat Flies Mirroring the Ecology of Their Hosts

**DOI:** 10.1371/journal.pone.0075215

**Published:** 2013-09-27

**Authors:** Pablo Tortosa, Najla Dsouli, Yann Gomard, Beza Ramasindrazana, Carl W. Dick, Steven M. Goodman

**Affiliations:** 1 Centre de Recherche et de Veille sur les Maladies Emergentes dans l’Océan Indien, Plateforme de Recherche CYROI, Ste Clotilde, La Réunion, France; 2 Université de La Réunion, Ste Clotilde, La Réunion, France; 3 Institut de Recherche pour le Développement, Sainte Clotilde, La Réunion, France; 4 Department of Biology, Western Kentucky University, Bowling Green, Kentucky, United States of America; 5 Field Museum of Natural History, Chicago, Illinois, United States of America; 6 Association Vahatra, Antananarivo, Madagasca; University of Milan-Bicocca, Italy

## Abstract

Bats and their parasites are increasingly investigated for their role in maintenance and transmission of potentially emerging pathogens. The islands of the western Indian Ocean hold nearly 50 bat species, mostly endemic and taxonomically well studied. However, investigation of associated viral, bacterial, and external parasites has lagged behind. In the case of their ectoparasites, more detailed information should provide insights into the evolutionary history of their hosts, as well as pathogen cycles in these wild animals. Here we investigate species of Nycteribiidae, a family of obligate hematophagous wingless flies parasitizing bats. Using morphological and molecular approaches, we describe fly species diversity sampled on Madagascar and the Comoros for two cave-roosting bat genera with contrasting ecologies: *Miniopterus* and *Rousettus*. Within the sampling area, 11 endemic species of insect-feeding *Miniopterus* occur, two of which are common to Madagascar and Comoros, while fruit-consuming *Rousettus* are represented by one species endemic to each of these zones. Morphological and molecular characterization of flies reveals that nycteribiids associated with *Miniopterus* bats comprise three species largely shared by most host species. Flies of *M*. *griveaudi*, one of the two bats found on Madagascar and certain islands in the Comoros, belong to the same taxon, which accords with continued over-water population exchange of this bat species and the lack of inter-island genetic structuring. Flies parasitizing *Rousettus* belong to two distinct species, each associated with a single host species, again in accordance with the distribution of each endemic bat species.

## Introduction

The bat faunas of western Indian Ocean islands provide a compelling context for natural history studies and host/parasites associations [1]. Islands of this region are among one of the 34 world biodiversity hotspots [2] and host nearly 50 bat species, and on Madagascar nearly 80% are endemic [3]. The phylogenetic and phylogeographic relations of much of the regional bat fauna, including species boundaries and distributional limits, are generally well resolved, while the description of potentially pathogenic microparasites has commenced [4], [5]. However, only fragmentary data are available concerning bat ectoparasites, which were targeted in the context of this study with the aim of understanding the level of host specificity and bat roosting associations as a window into their evolutionary history.

Two groups of bats were investigated in this study, which occur on Madagascar and on islands in the Comoros Archipelago (Malagasy Region) but differ in their ecology. Species of *Rousettus* (Pteropodidae) are frugivorous, while species of *Miniopterus* (Miniopteridae) are insectivorous, and members of these genera can often occur in close proximity in the same cave day-roosts. In our sample, *Rousettus* is represented by two distinct species showing a sister-species relationship: 1) *R*. *madagascariensis*, which has a metapopulation structure across Madagascar [6], and 2) *R*. *obliviosus*, which occurs in the Comoros but shows little phylogeographic structure with respect to the islands of Grande Comore (Ngazidja), Anjouan (Ndzuani), and Mohéli (Mwali) [6]. The geographical ranges of *R*. *madagascariensis* and *R*. *obliviosus* are separated by an over-water distance of <300 km, almost equal in distance to the nearest continental African member of this genus, *R*. *aegyptiacus*. The relationships of *R*. *madagascariensis* and *R*. *obliviosus* with respect to other African and Asian members of the genus remain unresolved.

Western Indian Ocean island species of *Miniopterus* have been the subject of detailed morphological and molecular analyses, and species limits are generally well documented. At certain day roost cave sites, up to four species co-occur [7]–[9], potentially facilitating cross-host transfer of ectoparasites. In addition, *M*. *griveaudi* and *M*. *aelleni* are shared between Madagascar and Comoros and based on bioacoustical, morphological, and genetic sequence data these populations show little intraspecific divergence [8]. The disjunctive nature of these island populations provides a means to test for possible host-parasite cospeciation. The monophyly of the Malagasy Region *Miniopterus* spp. has yet to be demonstrated.

Members of the regional bat fauna are host to numerous ectoparasite lineages, including mites, fleas, ticks, and bat flies. In the context of this study, we focused on bat flies of the family Nycteribiidae because (i) the ecology of these wingless flies is expected to encourage high host specificity and be informative about the evolutionary history of their hosts, and (ii) they parasitize the majority of regional bat species, allowing insight into fly diversity. We conducted an investigation based on morphological and molecular characterization (mitochondrial and nuclear markers) of bat flies on Madagascar and in the Comoros, with the intent to document aspects of host specificity and potential aspects of coevolution, as well as to compare and contrast aspects of differing ecology of the hosts to explain some of the observed patterns. Data presented herein on nycteribiid flies, overlaid on explicit phylogenies of their bat hosts, and together with other current investigations of various parasites of these animals, will provide a detailed view into the evolutionary history of these regional volant mammals in an insular context.

## Materials and Methods

### Taxon Sampling and Morphological Identification

Bats were captured on Madagascar and in the Union of the Comoros using mist nets and harp traps most often placed at cave entrances, or butterfly nets to obtain individuals from cave roost sites. This study was conducted in strict accordance with the terms of research permits issued by national authorities (Direction du Système des Aires Protégées, Direction Générale de l’Environnement et des Forêts, and Madagascar National Parks [Madagascar]; and Centre National de Documentation et de Recherche Scientifique [Union of the Comoros]), following the laws of these countries, and the associated research permit numbers are listed in the acknowledgements. Upon capture, each individual bat was placed in a separate clean cloth bag until the collection of relevant biological data, which included species, age and sex. Further, the GPS coordinates and other details on the capture sites were noted. Each bat specimen was systematically examined for ectoparasites, which were collected with forceps and stored in separate vials containing 70% ethanol. Animals were captured, manipulated, and dispatched with thoracic compression in accordance with guidelines accepted by the scientific community for the handling of wild mammals [10]. Morphological identifications of the ectoparasites were carried out using published keys and descriptions [11] and by reference to specimens housed in the Field Museum of Natural History (FMNH), Chicago. Molecular barcodes were generated for each fly by extracting DNA from whole specimens or a single intermediate leg, and sequencing mitochondrial Cytochrome Oxidase subunit I (COI) and nuclear 18S encoding gene as detailed below. Bat voucher specimens were identified using morphological and genetic characters and deposited in the FMNH and the Université d’Antananarivo, Département de Biologie Animale (UADBA), Antananarivo (Table S1 in Tables S1).

### Extraction and Amplification

Whole specimens or single intermediate legs were crushed using 3 mm tungsten beads (2 beads per specimen) in a Qiagen Tissue Lyser (Qiagen, Valencia, CA). DNA was extracted using a Qiagen EZ1 robot with DNA Tissue Kit according to the manufacturer’s protocol. Amplification of the COI was performed with barcoding LCO1490 and HCO2198 primers [12]. Amplification of nuclear 18S ribosomal DNA was carried out with the following primers: 1.2F, 18Sai, 18S b0.5, and 18S 7R [13]. All polymerase chain reactions (PCR) were conducted in 25 µl reaction mixtures containing 12.5 µl of GoTaq® Hot start green master mix (Promega, Madison, WI), 0.4 µM of each primer set and 1 µl of extracted DNA. The amplification profile was 94°C for 4 min, followed by 40 cycles of 40 s at 94°C, 40 s at an optimal annealing temperature (50°C and 57°C for LCO/HCO and 1.2F/7R primer sets, respectively), and 60 s at 72°C. PCR was followed by a final extension step of 10 min at 72°C. PCR products were separated by electrophoresis on 2% agarose gel, stained with 1X GelRed™ (Biotium Inc.), and visualized under UV light. PCR products were sequenced at GATC Biotech (Konstanz, Germany) using both forward and reverse primers.

### Nycteribiidae Phylogenetic Analyses

All sequences were automatically aligned using MAFFT implemented in Geneious Pro version 5.3.4 [14]. Multiple sequence alignments were subsequently adjusted by visual inspection, using as a reference nycteribiid species sequences available on GenBank. COI sequences were easily aligned as no insertions or deletions were detected. 18S alignments were cleaned from problematic alignment blocks using Gblocks 0.91 [15] with the following parameters: minimum number of sequences for a conserved position = 38; minimum number of sequences for a flanking position = 38; maximum number of contiguous non-conserved positions = 8; minimum length of a block = 5; allowed gap positions = with half.

Phylogenies were constructed separately for mitochondrial (COI) and nuclear (18S) datasets using homologous sequences available in GenBank as outgroups and ingroups (Table S1 in Tables S1). The Incongruence Length Difference (ILD) implemented in Paup 4.0b10 [16] was used to test the congruence of both markers. The best-fitting model and associated parameters were selected by jModelTest [17].

Phylogenies were constructed by Bayesian inference using MrBayes 3.1.2 [18]. The analysis consisted of two independent runs of four incrementally heated Metropolis Coupled Markov Chain Monte Carlo (MCMCMC) starting from a random tree. MCMCMC was run for 2,000,000 generations with trees and associated model parameters sampled every 300 generations. The initial 2000 trees were discarded as burn-in and the harmonic mean of the likelihood was calculated by combining the two independent runs. The 50% majority-rule consensus tree was then computed from the sampled trees in the two independent runs under the best model.

### Host Phylogeny and Cophylogenetic Analysis

We used ParaFit in order to test potential coevolution between nycteribiid flies and their bat host species [19]. Implemented test used a matrix of parasites/hosts associations (absence/presence of a parasite species on a bat species) together with two matrixes of patristic distances obtained from the reconstructed phylogenies of parasites and hosts. We used the nycteribiid COI generated phylogeny and bat species cytochrome *b* (*cyt b)* phylogeny based on available GenBank sequences (Table S2 in Tables S1) according to the best model of sequence evolution proposed by jModelTest [17]. Then, the two matrixes patristic distances were obtained with Patristic software [20]. Finally, the test was performed using ParaFit implemented in the package APE version 3.0–6 [21] under R software version 2.15.1 [22].

### Divergence Time Estimates

Based on COI, fly clade divergence times were estimated with BEAST v.1.7.1 [23] using a strict molecular clock and the standard mutation rate of arthropods of 1.15×10^−8^ nucleotide substitutions site^−1^ year^−1^ [24]. Well-supported nodes determined in the present mitochondrial phylogeny were enforced in BEAST before analysis. Analyses consisted of two runs of 20 million generations with sampling every 1000 generations. The initial 10% of trees from each run were discarded as burn-in. We used LogCombiner v.1.7.1 to combine both runs and analyzed the results with Tracer v.1.5. Convergence was confirmed by Effective Sample Size (ESS) values exceeding 200 for all parameters. A consensus chronogram of the maximum sum of clade support was obtained with TreeAnnotater v.1.7.1.

## Results

### Fly Morphological Diagnosis and Host Specificity

**Figure 1:**
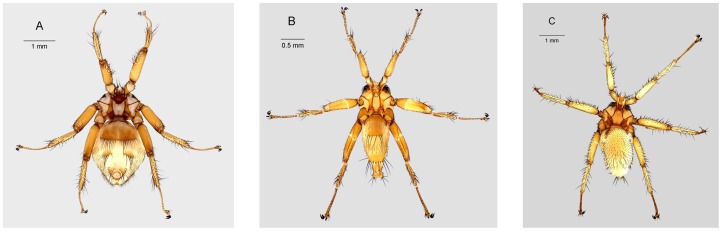
Representative genera of nycteribiid bat flies from Madagascar. A) *Penicillidia leptothrinax*, adult female, dorsal habitus, from *Miniopterus manavi*; B) *Nycteribia stylidiopsis*, adult female, dorsal habitus, from *M*. *gleni*; C) *Eucampsipoda madagascarensis*, adult female, dorsal habitus, from *Rousettus madagascariensis*.

The nycteribiid bat flies collected from species of *Rousettus* and *Miniopterus* belonged to three genera - *Eucampsipoda*, *Nycteribia*, and *Penicillidia* (Table 1, Fig. 1). Species of *Eucampsipoda* were limited to *Rousettus*, with two species, *E*. *madagascarensis* and *E*. *theodori* sampled from *R*. *madagascariensis* and *R*. *obliviosus*, respectively. Nycteribiid flies obtained from species of Miniopteridae included *Nycteribia stylidiopsis*, *Penicillidia* sp., and *P. leptothrinax*. *Nycteribia stylidiopsis* were sampled from multiple bat species – on Madagascar: *Miniopterus aelleni*, *M*. *gleni*, *M*. *majori*, and *M*. *petersoni*, and on Madagascar and the Comoros: *M*. *griveaudi*. Similarly, *P*. *leptothrinax* were sampled from *M*. *gleni*, *M*. *griveaudi* (Madagascar only), *M*. *mahafaliensis*, *M. majori, M*. *manavi*, *M*. *petersoni*, and *M*. *sororculus*. *Penicillidia* sp. parasitized *M*. *griveaudi* (Madagascar only) and *M*. *gleni*. In one case, *P*. *leptothrinax* and *N*. *stylidiopsis* were sampled on the same individual of *M*. *petersoni*.

**Table 1:** Nycteribiidae species identified from different bat species of the genera *Rousettus* and *Miniopterus* on Madagascar and the *Comoros archipelago and belonging to the genera *Eucampsipoda*, *Nycteribia*, and *Penicillidia*.

Bat hosts		Ectoparasitic nycteribiid flies				
Family	Species	*E*. *madagascarensis*	*E*. *theodori*	*N*. *stylidiopsis*	*P*. *leptothrinax*	*P*. sp.
Pteropodidae	*R*. *madagascariensis*	+	−	−	−	−
	*R*. obliviosus*	−	+	−	−	−
Miniopteridae	*M*. *aelleni*	−	−	+	−	−
	*M. gleni*	−	−	+	+	+
	M. griveaudi*	−	−	+	−	−
	*M. griveaudi*	−	−	+	+	+
	*M*. *mahafaliensis*	−	−	−	+	−
	*M*. *majori*	−	−	+	+	−
	*M*. *manavi*	−	−	−	+	−
	*M*. *petersoni*	−	−	+	+	−
	*M*. *sororculus*	−	−	−	+	−

+/−: presence/absence of a given parasite on the bat species.

### Molecular Diagnosis accords with Morphological Identification

The morphology-based identifications were compared to a molecular phylogeny obtained with the nuclear and mitochondrial markers. The most suitable substitution models based on Akaike Information Criterion were GTR+I+G and HKY+I+G for both mitochondrial and nuclear data, respectively. Amplification of mitochondrial COI produced 44 sequences of 658 bp and the amplification of the nuclear 18S locus produced 45 sequences ranging from 826 to 2139 bp. All sequences are available on GenBank under accession numbers KF021491 to KF021528 for mitochondrial haplotypes, and under accession numbers KF156670 to KF156714 for nuclear haplotypes (Table S1 in Tables S1).

**Figure 2:**
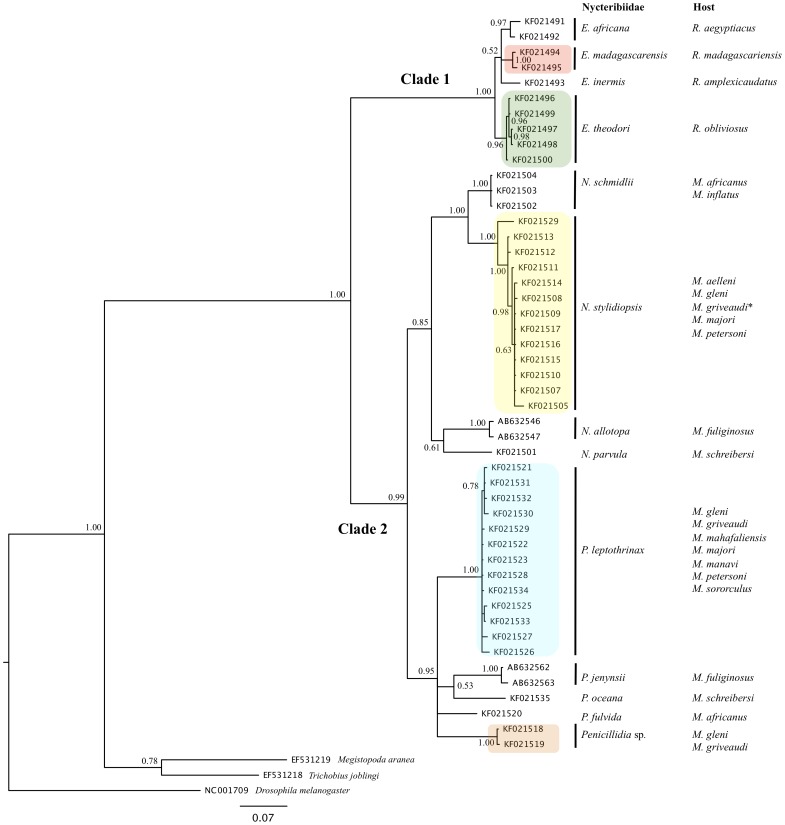
Phylogenetic tree based on mitochondrial sequences (COI). Nycteribiid flies were sampled from bats collected in Madagascar (*Miniopterus aelleni*, *M*. *gleni*, *M*. *griveaudi*, *M*. *mahafaliensis*, *M*. *majori*, *M*. *manavi*, *M*. *petersoni*, and *M*. *sororculus*) and Comoros (*Rousettus obliviosus* and *M*. *griveaudi*). The analysis was carried out using Bayesian Inference under the GTR+I+G substitution model, and nodal support values reflect Bayesian Probabilities. (*) indicates *M*. *griveaudi* sampled on Madagascar and Comoros.

The COI*-*based phylogeny comprises two distinct clades (1 and 2), both supported by posterior probabilities (P.P) of 1.00 and 0.99, respectively (Fig. 2). Clade 1 comprises species of *Eucampsipoda*, while clade 2 includes two subclades, one each for species of *Nycteribia* (P.P: 0.85) and *Penicillidia* (P.P: 0.95), respectively (Fig. 2). Morphology-based systematics agrees with COI-based topology depicting two distinct Malagasy/Comoros species within clade 1. With the inclusion of sequences from two fly species, *E*. *africana* and *E*. *inermis*, parasitizing African *Rousettus aegyptiacus* and Asian *R*. *amplexicaudatus*, respectively, this pattern was maintained. In the case of *P*. *leptothrinax* and *N*. *stylidiopsis* obtained from Madagascar and Comorian populations of *Miniopterus griveaudi*, no intra-specific difference was found related to the geographical origin of the samples (Fig. 2).

### Nuclear and Mitochondrial Phylogenies are Congruent

**Figure 3:**
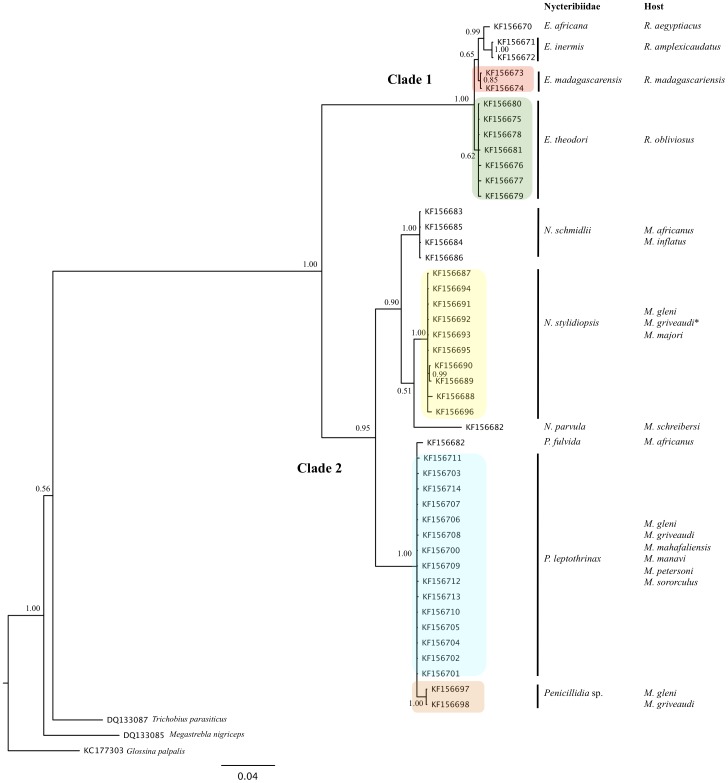
Phylogenetic tree based on nuclear sequences (18S). Nycteribiid flies were sampled from bats collected in Madagascar (*Miniopterus aelleni*, *M*. *gleni*, *M*. *griveaudi*, *M*. *mahafaliensis*, *M*. *majori*, *M*. *manavi*, *M*. *petersoni*, and *M*. *sororculus*) and Comoros (*Rousettus obliviosus* and *M*. *griveaudi*). The analysis was carried out using Bayesian Inference under the HKY+I+G substitution model. Nodal support values reflect Bayesian Probabilities.

The topology obtained with the nuclear marker is similar to that of the mitochondrial marker, and displays two main clades, clade 1 and clade 2, supported by PP = 1.00 and PP = 0.95 respectively (Fig. 3). Clade 1 comprises the two groups corresponding to *E*. *madagascarensis* and *E*. *theodori* sampled from *Rousettus madagascariensis* and *R*. *obliviosus*, respectively, and clade 2 comprises members of the genera *Nycteribia* (P.P: 0.90) and *Penicillidia* (P.P: 1.00) obtained from *Miniopterus* spp. Within *Nycteribia*, *N*. *stylidiopsis* forms a monophyletic group (P.P: 1.00) including flies collected in the Comoros and Madagascar. Similarly, the *Penicillidia* clade includes *P*. *leptothrinax* and *P*. sp. Overall, our data indicate that Madagascar and Comoros nycteribiid flies parasitizing *Rousettus* comprise two distinct taxa, each host specific and with disjunct distributions. Among bat flies of *Miniopterus* spp., three distinct taxa can be recognized and for two of these, there is no separation of Comorian and Malagasy populations and no clear division based on host species. Altogether, molecular phylogenies obtained with nuclear and mitochondrial markers are congruent (ILD test, *P*>0.05) and, with the exception of showing African *P*. *fulvida* as distinct from Malagasy *P*. sp., confirm the previously proposed species status of these flies based on morphological identification.

### Estimation of Nycteribiidae Divergence Times

Figure S1 depicts divergence times estimated for lineages of nycteribiid species parasitizing African and Asian bats, using the standard mutation rate of arthropods and COI mitochondrial sequences. The deepest node (10.88 Myr, 95% CI: 8.57–13.33) among Malagasy and Comorian flies segregates species parasitizing Miniopteridae and Pteropodidae regardless of the African or Asian ancestry of these taxa. The origin of flies associated with Miniopteridae is dated at 6.98 Myr (95% CI: 5.69–8.42); once again, Asian and African flies are imbedded in the same cluster. According to the analysis, *E*. *theodori* and *E*. *madagascarensis* diverged 1.61 Myr (95% CI: 1.10–2.15).

## Discussion

Bat flies, particularly New World members of the family Streblidae, are generally understood to be host specific, with one fly species typically parasitizing one host species or a few closely related host species [25], [26]. This high degree of host specificity is remarkable in light of life history traits and reproductive strategies of bat flies [27]. Female bat flies, like other hippoboscoid Diptera, rear a single offspring at a time. All three larval stages develop internally, and a single puparium is deposited away from the host bat and inside the roosting environment [28]. An exception to this general pattern is found in the little-known Old World subfamily Ascodipterinae, where adult females become endoparasitic and do not leave their host to deposit pupae [29]. For nycteribiid flies, the pupal stage lasts 3–4 weeks [30], [31], and then the newly emerged adult fly must seek, find, and colonize a host bat. This decoupling of flies from their hosts, particularly in day-roosts holding multiple species of bats, should discourage host specificity among bat flies. Further, all nycteribiids are wingless, whereas ∼85% of streblids possess functional wings [28]. Dispersal limitations of nycteribiids relative to streblids should work to increase host specificity of the former. Although factors such as prevalence (proportion of host population parasitized) and intensity (average number of parasites per host) of parasitism by bat flies appear to be high in cave day-roosts [32], the degree that host specificity varies based on roost type or relative distances between the roosts in the same cave of different bat species is unknown. Moreover, the degree to which nycteribiid species are host specific is little studied, though Marshall reported that the majority of Malaysian nycteribiids were limited to a single host species [33].

Although ectoparasitic bat flies of the families Streblidae and Nycteribiidae are subjects of increasing research, the present study is to our knowledge the first to elucidate phylogenetic relationships among species of nycteribiids parasitizing frugivorous and insectivorous bats. The western Indian Ocean islands are well suited for the investigation of these host-parasite associations as they harbor an important diversity of mostly endemic bat species with different species assemblages across the region. Previous genetic research suggested that *R*. *madagascariensis* and *R*. *obliviosus* are distinct sister-taxa and limited in distribution to Madagascar and Comoros (Grande Comore, Anjouan, and Mohéli), respectively, with no evidence of gene flow between populations of these species, separated by an over water distance of at least 300 km [6]. Conversely, *Miniopterus* is diverse, with 11 currently recognized species occurring on Madagascar. On the basis of bioacoustical and *cyt b* genetic data, it has been confirmed that two of these species, *M*. *griveaudi* and *M*. *aelleni*, also occur on Grande Comore and Anjouan and these populations are derived from Malagasy colonizers [8].

Our sampled bats show contrasting patterns of ectoparasitism: each of the two western Indian Ocean *Rousettus* spp. is parasitized by a distinct nycteribiid species, whereas eight parasitized *Miniopterus* spp. share three fly species. Flies found on *M*. *griveaudi* sampled on Madagascar and the Comoros are morphologically similar, suggesting no differentiation among flies of the two island groups; this is supported by molecular analyses carried out with COI and 18S markers. The phylogenies obtained with both mitochondrial and nuclear markers are congruent, thus lowering the probability of introgression that may contribute to phylogenetic misinterpretation [34], [35].

The fly phylogeny depicts one clade containing all flies parasitizing African and Asian *Rousettus* and one clade with all species parasitizing Asian and African *Miniopterus* spp. *Eucampsipoda theodori*, specific to *R*. *obliviosus* from the Comoros, is clearly distinct from other species parasitizing Asian, Malagasy or African members of this bat genus, which may result of geographical isolation of *R*. *obliviosus*. The resolution of our phylogeny does not allow inference of an African or Asian origin of *R*. *madagascariensis* and its sister-species *R*. *obliviosus*.

Flies parasitizing *Miniopterus* spp. show low host specificity, with specimens sampled on the Comoros and Madagascar exhibiting almost identical haplotypes. The lack of host specificity for *Nycteribia stylidiopsis* and *Penicillidia leptothrinax* species (See Table 1 and Fig. S2) suggests multiple host switches. In certain cases, both fly species were sampled from the same individual of *M*. *petersoni*, suggesting coexistence of these two fly species on individual bats. In light of nycteribiid life-history traits mentioned above and the mixed assemblages of different *Miniopterus* spp. at the same roost sites (Table 1), this result is not unexpected. Little is known about migratory movements of Malagasy *Miniopterus* spp., but for widespread taxa, such as *M*. *griveaudi* and *M*. *gleni*, there is no pronounced phylogeographical structure [7], [36], [37]. Hence, it can be assumed that these bat species undergo considerable dispersal, which also may explain the lack of nycteriibid host specificity and geographical structure. However, there appear to be notable limits to potential host switching. Day roosts of *Rousettus* and *Miniopterus* in both the Comoros and Madagascar occur in the same caves with the physical possibility of bat fly exchange between these genera (Table 2). Yet we found no evidence of *Eucampsipoda* on *Miniopterus*, or *Nycteribia*, *Penicillidia*, and *Nycteribia* on *Rousettus*. However, different *Miniopterus* spp., which may be found in close proximity or similar distances to one another as compared to *Rousettus* roosts, are clearly exchanging bat ectoparasites.

**Table 2:** Known cave roosting associations of bat species of the genera *Rousettus* and *Miniopterus* (this study).

Species Species	Sympatric occurrence in cave roost
*R*. *madagascariensis*	*M*. *aelleni*, *gleni*, *griveaudi*
*R*. obliviosus*	[*M*. *aelleni*], *griveaudi*
*M*. aelleni*	[*M*. *griveaudi*], [*R*. *obliviosus*]
*M*. *aelleni*	*M. gleni*, *griveaudi*, *R*. *madagascariensis*
*M*. *gleni*	*M*. *aelleni*, *griveaudi*, [*mahafaliensis*],*R*. *madagascariensis*
*M*. griveaudi*	[*M*. *aelleni*], *R*. *obliviosus*
*M*. *griveaudi*	*M*. *aelleni*, *gleni*, *R*. *madagascariensis*
*M*. *mahafaliensis*	[*M*. *gleni*], *R*. *madagascariensis*
*M*. *majori*	*M. manavi*, *soroculus*
*M*. *manavi*	*M*. *majori*, *sororculus*, [*R*. *madagascariensis*]
*M*. *petersoni*	*M*. *majori*, [*R*. *madagascariensis*]
*M*. *sororculus*	*M*. *mahafaliensis*, *majori*

Data from field inventories of species assemblages documented at different sites on Madagascar and the *Comoros archipelago. Names presented in brackets are inferred sympatric occurrences based on capture at a site in close proximity.

The estimated divergence time of fly clades can be overlaid upon the geological history of regional landmasses in order to examine different diversification scenarios for these insular species: Madagascar originated from Gondwanan drift (88–130 Myr), while the Comoros Archipelago (0.13–15 Myr) is composed of oceanic volcanic islands formed *de novo* [38], [39]. A previous study carried out on both western Indian Ocean *Rousettus* spp. proposed an isolation date between these species of 0.358 Myr (90% HPD = 0.229–0.582 Myr) [6]. Our estimation using the average mutation rate of arthropods suggests a divergence between *Eucampsipoda theodori* and *E*. *madagascarensis* occurring 1.61 Myr (95% CI: 1.10–2.15) ago, thus prior to the estimated divergence of their *Rousettus* hosts [6]. These data suggest that flies may have diverged earlier than hosts, although comparison of vertebrate and invertebrate divergence estimates requires cautious interpretation. Nonetheless, this estimation is older than the emergence dates of Grande Comore, one the three islands in the Comoros Archipelago where *R*. *obliviosus* is known to occur, [38], [39]). Such timing would be compatible with the isolation of *R*. *obliviosus* ancestral populations on Anjouan, and this island serving as a source for subsequent colonization of Grande Comore and Mohéli. Alternatively, the ancestral *E*. *theodori* might have diverged on Madagascar or continental Africa before colonizing Comoros and went extinct in the area of origin.

Although *Rousettus* and *Miniopterus* species have distinct ecologies (including diet), it is important to note that these bats often have day roost sites in the same caves (see Table S2 in Tables S1). In some cases, roosts can be composed of mixed species of *Miniopterus* in close physical proximity. Thus, host structuring in the roosting sites alone may not explain the contrasting patterns of bat fly host specialization and host switching as noted above. By contrast, the similarity of bat (at least at the generic level) and fly phylogenies (Fig. S2) as well as the estimation of bat flies divergence times support a specialization resulting from coevolutionary processes. This was tested comparing *cyt b* phylogeny of bats and COI phylogeny of flies using ParaFit. However, our data set could not reject the null hypothesis of absence of coevolution (P>0.05), which might in part be associated with the lack of resolution in portions of the *Miniopterus* phylogeny.

Altogether, nycteribiid phylogenies are at certain levels consistent with the evolutionary history of *Miniopterus* and *Rousettus* spp. occurring on western Indian Ocean islands (Fig. S2), suggesting in some cases coevolution; our data are insufficient to support explicitly this hypothesis. As the phylogeny of Old World *Rousettus* remains unresolved and there is evidence of considerable paraphyly in African *Miniopterus* [40], these aspects will need to be resolved before further tests of coevolution can be properly employed. In any case, our results show that ectoparasites investigated herein range from host specialists to generalists, and this should be taken into consideration when investigating the diversity and dynamics of bat fly ectoparasites in western Indian Ocean bats.

## Supporting Information

Figure S1 **Estimation of divergence times of nycteribiid lineages.** Chronogram based on Bayesian analysis of mitochondrial sequences (COI) of bat fly species from Madagascar (*Eucampsipoda madagascarensis*, *Penicillidia leptothrinax*, *P*. *fulvida*, *P*. sp, and *Nycteribia stylidiopsis*), the Comoros (*E*. *theodori* and *N*. *stylidiopsis*), continental Africa (*E*. *africana* and *N. schmidlii*) and Asia (*E*. *inermis*, *N*. *allotopa*, *N. parvula, P. jenynsii*, and *P*. *oceana*). Other species were used as outgroups: *Drosophila melanogaster*, *Trichobius joblingi*, and *Megistopoda aranea*. The values indicate the estimated average divergence time in million years (Myr) based on the general mutation rate of arthropods (1.15×10^−8^ nucleotide substitutions site^−1^ year^−1^). The 95% confidence intervals and posterior probabilities are indicated.
(TIFF)

Figure S2 **Reconciliation trees of bats and nycteribiid bat flies.** Bat species trees were constructed using Phyml with 100 replicates and *cyt b* sequences (GenBank accession numbers provided in Table S2). Nycteribiidae tree was generated similarly, using COI sequences (this study) and COI sequences available on GenBank (see Table S1 in Supporting Tables file).
(TIFF)

Tables S1 **Includes Table S1 and S2.** Table S1. Details of specimens used in the present study: GenBanK accession numbers, host, and origin. Table S2. Details of bats *cyt b* sequences used in this study with their GenBanK accession numbers.
(DOC)

Permits S1 (PDF)
