# Supplementary figures and images for: Evolutionary History of Indian Ocean Nycteribiid Bat Flies Mirroring the Ecology of Their Hosts
Source: PLoS One. 2013 Sep 27;8(9):e75215. doi: 10.1371/journal.pone.0075215 (PMC3785519; doi:10.1371/journal.pone.0075215)

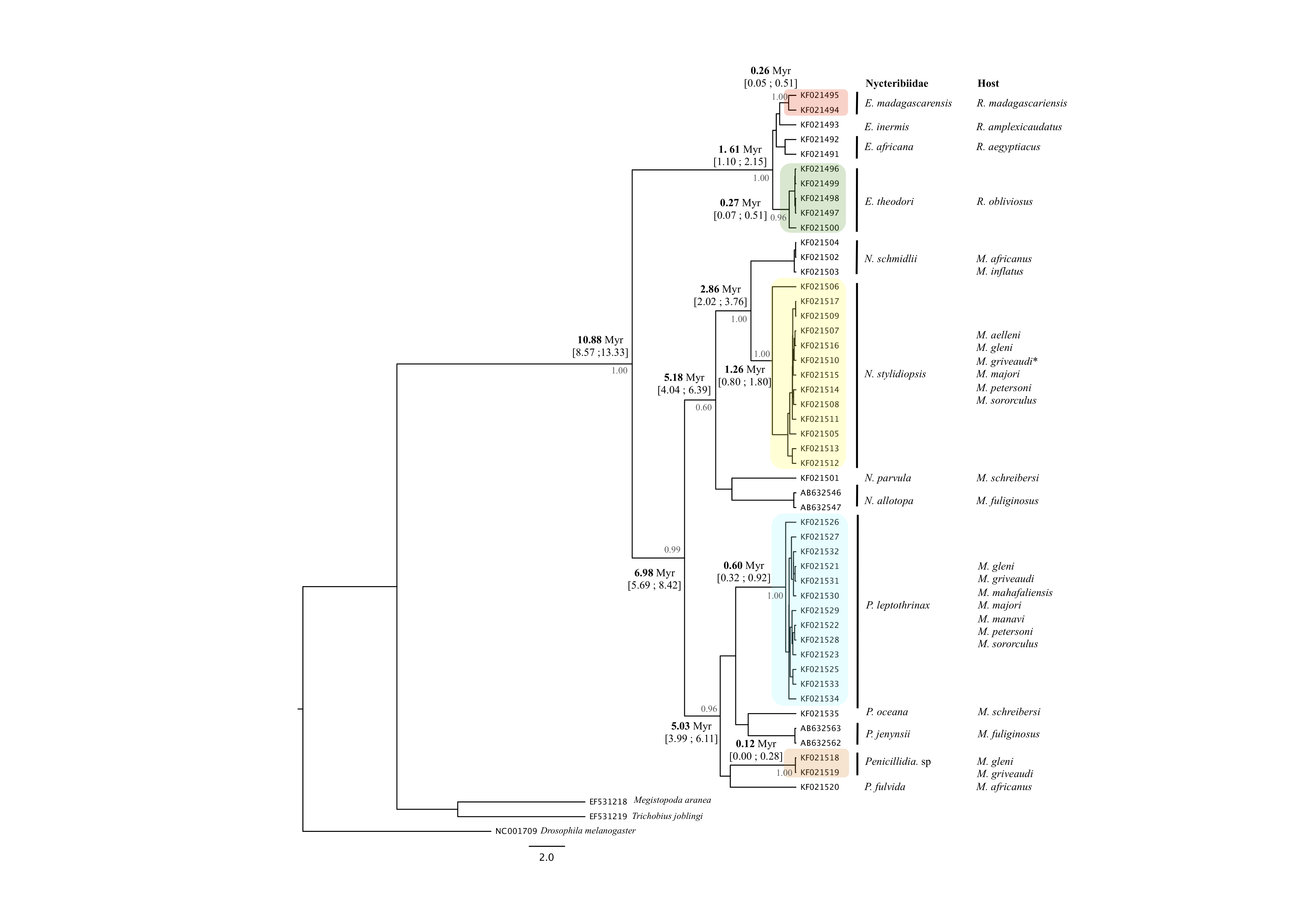

Supplement: Figure S1 — Estimation of divergence times of nycteribiid lineages. Chronogram based on Bayesian analysis of mitochondrial sequences (COI) of bat fly species from Madagascar (Eucampsipoda madagascarensis, Penicillidia leptothrinax, P. fulvida, P. sp, and Nycteribia stylidiopsis), the Comoros (E. theodori and N. stylidiopsis), continental Africa (E. africana and N. schmidlii) and Asia (E. inermis, N. allotopa, N. parvula, P. jenynsii, and P. oceana). Other species were used as outgroups: Drosophila melanogaster, Trichobius joblingi, and Megistopoda aranea. The values indicate the estimated average divergence time in million years (Myr) based on the general mutation rate of arthropods (1.15×10^−8^ nucleotide substitutions site−1 year−1). The 95% confidence intervals and posterior probabilities are indicated. (TIFF) [file pone.0075215.s001.tif]

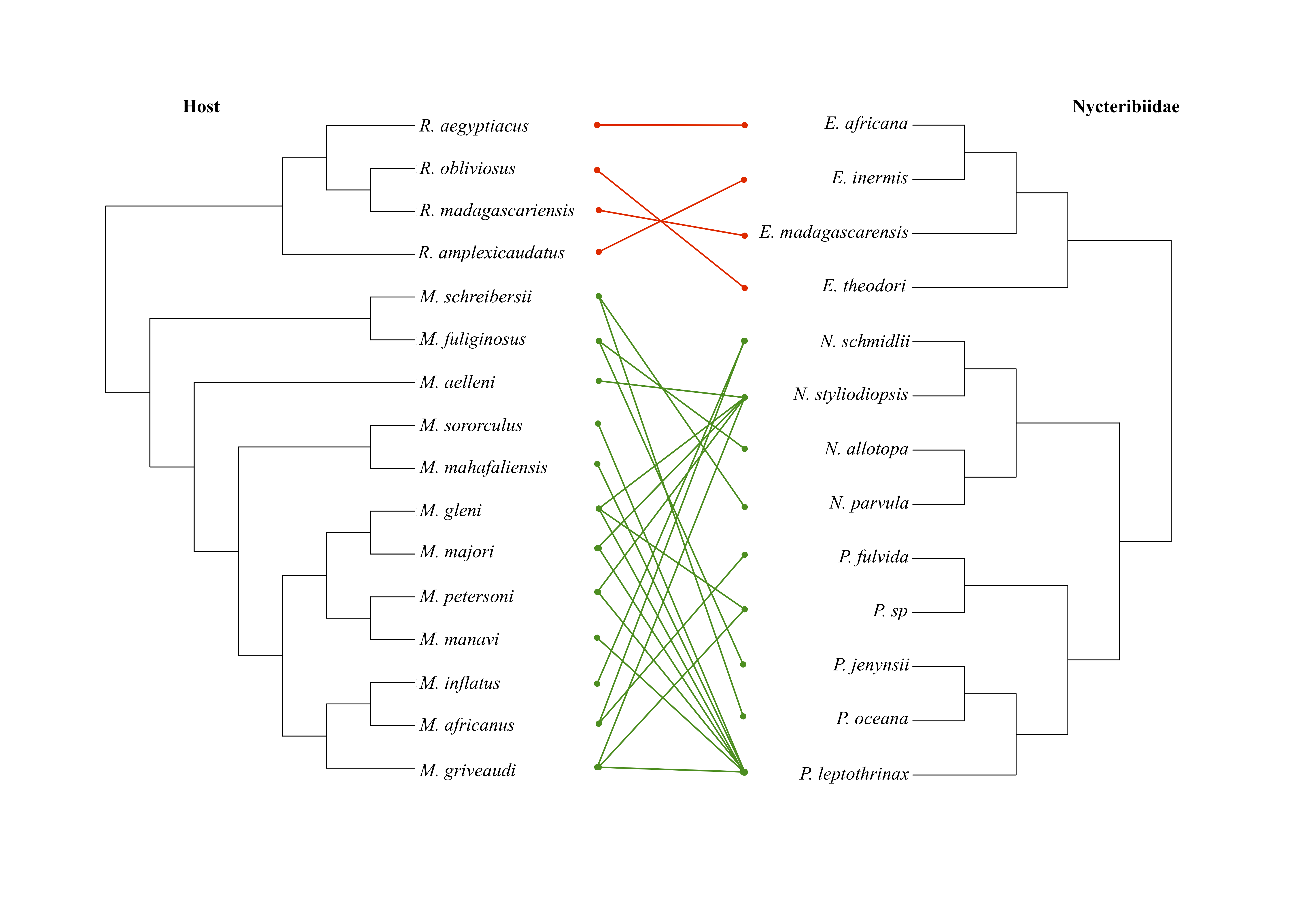

Supplement: Figure S2 — Reconciliation trees of bats and nycteribiid bat flies. Bat species trees were constructed using Phyml with 100 replicates and cyt b sequences (GenBank accession numbers provided in Table S2). Nycteribiidae tree was generated similarly, using COI sequences (this study) and COI sequences available on GenBank (see Table S1 in Supporting Tables file). (TIFF) [file pone.0075215.s002.tiff]
