# Supplementary material for: Evolutionary History of Indian Ocean Nycteribiid Bat Flies Mirroring the Ecology of Their Hosts
Source: PLoS One. 2013 Sep 27;8(9):e75215. doi: 10.1371/journal.pone.0075215 (PMC3785519; doi:10.1371/journal.pone.0075215)
Supplement: Tables S1 — Includes Table S1 and S2. Table S1. Details of specimens used in the present study: GenBanK accession numbers, host, and origin. Table S2. Details of bats cyt b sequences used in this study with their GenBanK accession numbers. (DOC) [file pone.0075215.s003.doc]

**Table S1.**

| **Nycteribiidae** | | **GenBank accession number** | | **Host** | | |
| --- | --- | --- | --- | --- | --- | --- |
| ***Species*** | ***Isolate*** | ***COI*** | ***18S*** | ***Species*** | ***Identity*** | ***Origin*** |
| *Eucampsipoda africana* | N4 | KF021491 | - | *Rousettus aegyptiacus* | BDP-5503 | Kenya |
| *Eucampsipoda africana* | N5 | - | KF156670 | *Rousettus aegyptiacus* | BDP-5503 | Kenya |
| *Eucampsipoda africana* | N6 | KF021492 | - | *Rousettus aegyptiacus* | BDP-4535 | Kenya |
| *Eucampsipoda inermis* | N8 | KF021493 | KF156671 | *Rousettus amplexicaudatus* | KU 165172 | Philippines |
| *Eucampsipoda inermis* | N9 | - | KF156672 | *Rousettus amplexicaudatus* | KU 164019 | Philippines |
| *Eucampsipoda madagascarensis* | J43 | KF021494 | KF156673 | *Rousettus madagascariensis* | UADBA 43757 | Madagascar |
| *Eucampsipoda madagascarensis* | J48 | KF021495 | KF156674 | *Rousettus madagascariensis* | FMNH 209105 | Madagascar |
| *Eucampsipoda theodori* | 16D | - | KF156679 | *Rousettus obliviosus* | FMNH SMG-16702 | Comoros |
| *Eucampsipoda theodori* | 18C | - | KF156680 | *Rousettus obliviosus* | FMNH SMG-16704 | Comoros |
| *Eucampsipoda theodori* | 24A | - | KF156681 | *Rousettus obliviosus* | FMNH SMG16710 | Comoros |
| *Eucampsipoda theodori* | 28B | - | KF156675 | *Rousettus obliviosus* | FMNH SMG-16714 | Comoros |
| *Eucampsipoda theodori* | 28DM | KF021496 | - | *Rousettus obliviosus* | FMNH SMG-16714 | Comoros |
| *Eucampsipoda theodori* | 29B | - | KF156676 | *Rousettus obliviosus* | FMNH SMG-16716 | Comoros |
| *Eucampsipoda theodori* | 29F | KF021497 | - | *Rousettus obliviosus* | FMNH SMG-16715 | Comoros |
| *Eucampsipoda theodori* | 30DM | KF021498 | - | *Rousettus obliviosus* | FMNH SMG-16716 | Comoros |
| *Eucampsipoda theodori* | 31A | - | KF156677 | *Rousettus obliviosus* | FMNH SMG-16751 | Comoros |
| *Eucampsipoda theodori* | 31B | - | KF156678 | *Rousettus obliviosus* | FMNH SMG-16751 | Comoros |
| *Eucampsipoda theodori* | 31DM | KF021499 | - | *Rousettus obliviosus* | FMNH SMG-16751 | Comoros |
| *Eucampsipoda theodori* | 8B | KF021500 | - | *Rousettus obliviosus* | FMNH SMG-16694 | Comoros |
| *Nycteribia parvula* | N16 | KF021501 | KF156682 | *Miniopterus schreibersi* | KU 165042 | Philippines |
| *Nycteribia schmidlii* | N21 | KF021502 | KF156683 | *Miniopterus africanus* | BDP-5665 | Kenya |
| *Nycteribia schmidlii* | N22 | - | KF156684 | *Miniopterus africanus* | BDP-5665 | Kenya |
| *Nycteribia schmidlii* | N2 | KF021503 | KF156685 | *Miniopterus inflatus* | BDP-5550 | Kenya |
| *Nycteribia schmidlii* | N3 | KF021504 | KF156686 | *Miniopterus africanus* | BDP-5600 | Kenya |
| *Nycteribia stylidiopsis* | 33A | KF021505 | KF156687 | *Miniopterus griveaudi* | SMG-16760 | Comoros |
| *Nycteribia stylidiopsis* | 42A | KF021506 | - | *Miniopterus petersoni* | FMNH 209186 | Madagascar |
| *Nycteribia stylidiopsis* | GR11 | - | KF156691 | *Miniopterus gleni* | FMNH SMG-17847 | Madagascar |
| *Nycteribia stylidiopsis* | GR12 | KF021507 | KF156688 | *Miniopterus gleni* | FMNH SMG-17846 | Madagascar |
| *Nycteribia stylidiopsis* | GR15 | - | KF156692 | *Miniopterus gleni* | UADBA 33030 | Madagascar |
| *Nycteribia stylidiopsis* | GR1 | - | KF156690 | *Miniopterus griveaudi* | FMNH SMG-17606 | Madagascar |
| *Nycteribia stylidiopsis* | GR2 | KF021508 | KF156689 | *Miniopterus griveaudi* | FMNH SMG-17616 | Madagascar |
| *Nycteribia stylidiopsis* | GR3 | KF021509 | - | *Miniopterus griveaudi* | UADBA 32944 | Madagascar |
| *Nycteribia stylidiopsis* | GR9 | KF021510 | KF156693 | *Miniopterus griveaudi* | FMNH SMG-17689 | Madagascar |
| *Nycteribia stylidiopsis* | J31 | KF021511 | - | *Miniopterus majori* | UADBA 43256 | Madagascar |
| *Nycteribia stylidiopsis* | J32 | KF021512 | KF156695 | *Miniopterus majori* | UADBA 43264 | Madagascar |
| *Nycteribia stylidiopsis* | J33 | KF021513 |  | *Miniopterus majori* | FMNH 209177 | Madagascar |
| *Nycteribia stylidiopsis* | J54 | - | KF156694 | *Miniopterus gleni* | UADBA 43897 | Madagascar |
| *Nycteribia stylidiopsis* | J60 | KF021514 | - | *Miniopterus aelleni* | FMNH 184065 | Madagascar |
| *Nycteribia stylidiopsis* | J61 | KF021515 | - | *Miniopterus griveaudi* | FMNH SMG-17612 | Madagascar |
| *Nycteribia stylidiopsis* | A | KF021516 | - | *Miniopterus gleni* | UADBA 43897 | Madagascar |
| *Nycteribia stylidiopsis* | B | KF021517 | KF156696 | *Miniopterus gleni* | UADBA 32373 | Madagascar |
| *Penicillidia* sp. | J68 | KF021518 | KF156697 | *Miniopterus gleni* | FMNH 218031 | Madagascar |
| *Penicillidia* sp. | GR21 | KF021519 | KF156698 | *Miniopterus griveaudi* | FMNH SMG-17753 | Madagascar |
| *Penicillidia fulvida* | N20 | KF021520 | KF156699 | *Miniopterus africanus* | BDP-5591 | Kenya |
| *Penicillidia leptothrinax* | GR7 | KF021521 | KF156700 | *Miniopterus griveaudi* | FMNH SMG-17674 | Madagascar |
| *Penicillidia leptothrinax* | GR18 | - | KF156709 | *Miniopterus griveaudi* | UADBA 33015 | Madagascar |
| *Penicillidia leptothrinax* | GR20 | - | KF156711 | *Miniopterus griveaudi* | FMNH SMG-17757 | Madagascar |
| *Penicillidia leptothrinax* | GR22 | KF021522 | - | *Miniopterus griveaudi* | FMNH SMG-17757 | Madagascar |
| *Penicillidia leptothrinax* | J62 | KF021523 | KF156701 | *Miniopterus manavi* | FMNH SMG-17880 | Madagascar |
| *Penicillidia leptothrinax* | 35A | KF021525 | - | *Miniopterus manavi* | FMNH 209179 | Madagascar |
| *Penicillidia leptothrinax* | 35B | KF021526 | KF156702 | *Miniopterus manavi* | FMNH 209179 | Madagascar |
| *Penicillidia leptothrinax* | 36A | KF021527 | - | *Miniopterus majori* | FMNH 209171 | Madagascar |
| *Penicillidia leptothrinax* | J70 | KF021528 | KF156703 | *Miniopterus sororculus* | UADBA SMG-17346 | Madagascar |
| *Penicillidia leptothrinax* | J71 | - | KF156710 | *Miniopterus mahafaliensis* | FMNH 217938 | Madagascar |
| *Penicillidia leptothrinax* | J72 | - | KF156712 | *Miniopterus sororculus* | UADBA SMG-17379 | Madagascar |
| *Penicillidia leptothrinax* | J73 | - | KF156713 | *Miniopterus sororculus* | FMNH SMG-17365 | Madagascar |
| *Penicillidia leptothrinax* | J34 | KF021529 | KF156704 | *Miniopterus petersoni* | FMNH 209186 | Madagascar |
| *Penicillidia leptothrinax* | J63 | KF021530 | KF156705 | *Miniopterus gleni* | UADBA SMG-17368 | Madagascar |
| *Penicillidia leptothrinax* | J64 | KF021531 | KF156706 | *Miniopterus gleni* | UADBA SMG-17373 | Madagascar |
| *Penicillidia leptothrinax* | J65 | KF021532 | KF156707 | *Miniopterus mahafaliensis* | FMNH 217933 | Madagascar |
| *Penicillidia leptothrinax* | J66 | KF021533 | KF156708 | *Miniopterus mahafaliensis* | FMNH 217936 | Madagascar |
| *Penicillidia leptothrinax* | J67 | KF021534 | KF156714 | *Miniopterus sororculus* | FMNH 217985 | Madagascar |
| *Penicillidia oceana* | N17 | KF021535 | - | *Miniopterus schreibersii* | KU 165154 | Philippines |
| *Megistopoda aranea* |  | EF531219 | - | Bats | - | - |
| *Trichobius joblingi* |  | EF531218 | - | Bats | - | - |
| *Drosophila melanogaster* |  | NC001709 | KC177303 | Not ectoparasitic | - | - |
| *Penicillidia jenynsii* |  | AB632562 | - | *Miniopterus fuliginosus* | - | Japan |
| *Penicillidia jenynsii* |  | AB632563 | - | *Miniopterus fuliginosus* | - | Japan |
| *Nycteribia allotopa* |  | AB632546 | - | *Miniopterus fuliginosus* | - | Japan |
| *Nycteribia allotopa* |  | AB632547 | - | *Miniopterus fuliginosus* | - | Japan |
| *Trichobius parasiticus* |  | - | DQ133087 | unknown | - | Mexico |
| *Megastrebla nigriceps* |  | - | DQ133085 | *Eonycteris spelaea* | - | Malaysia |

**Table S2.**

| **Family** | **Species** | **GenBank accession number** | **Origin** |
| --- | --- | --- | --- |
| Pteropodidae | *Rousettus amplexicaudatus* | AB046327 | Philippines |
|  | *Rousettus aegyptiacus* | JX274497 | Egypt |
|  | *Rousettus obliviosus* | GU228753 | Comoros |
|  | *Rousettus madagascariensis* | GU228684 | Madagascar |
| Miniopteridae | *Miniopterus schreibersii* | AY208140 | Japan |
|  | *Miniopterus fuliginosus* | AB085735 | Japan |
|  | *Miniopterus inflatus* | AY614737 | Africa |
|  | *Miniopterus mahafaliensis* | JF440264 | Madagascar |
|  | *Miniopterus petersoni* | FJ383131 | Madagascar |
|  | *Miniopterus manavi* | JF440280 | Madagascar |
|  | *Miniopterus gleni* | JF440234 | Madagascar |
|  | *Miniopterus griveaudi* | JF440255 | Madagascar |
|  | *Miniopterus majori* | JF440270 | Madagascar |
|  | *Miniopterus africanus* | EF363524 | Africa |
|  | *Miniopterus sororculus* | DQ899773 | Madagascar |
|  | *Miniopterus aelleni* | JF440219 | Madagascar |
