# Supplementary material for: Evolutionary History of Indian Ocean Nycteribiid Bat Flies Mirroring the Ecology of Their Hosts
Source: PLoS One. 2013 Sep 27;8(9):e75215. doi: 10.1371/journal.pone.0075215 (PMC3785519; doi:10.1371/journal.pone.0075215)
Supplement: Permits S1 — (PDF) [file pone.0075215.s004.pdf]

REPOBLIKAN'I MADAGASIKARA
Fitiavana-Tanindrazana -Fandrosoana

MINISTERE DE L'ENVIRONNEMENT ET DES FORETS
B.P: 610, Rue Fernand Kasanga – Tsimbazaza ANTANANARIVO – 101-
Tel: (261 20) 22 668 05 – Fax: (261 20) 22 354 10

AUTORISATION DE :

x - RECHERCHE
- ETUDE

N° 283 /11/MEF/SG/DGF/DCB.SAP/SCB
NOM RAMASINDRAZANA
PRENOMS Beza
ADRESSE B.P : 906 Antananarivo
FONCTION Chercheur
ACCOMPAGNE DE : Steven Goodman, un représentant du CAFF/CORE.

ORGANISME TUTELLE : Département de Biologie Animale (DBA)

EST AUTORISE(E) A FAIRE DES RECHERCHES / ETUDES DANS
Zone de Ranohira et Ilakaka en dehors du P.N d'Isalo.
« L'accès dans les forêts ayant un gestionnaire et/ou transférées à un comité de gestion fera l'objet de négociation avec ce dernier »

MENTION SPECIALE EVENTUELLE:
Inventaire de la faune chiroptérologique
Capture avec relâche de chauves –souris de la famille des *Vespertilionidae* pour études morphologique, morphométrique et bioacoustique
Collecte d'au maximum deux individus par espèce pour identification
Prélèvement d'échantillons de tissu pour les les études moléculaires.

DUREE : Un (01) mois.

N.B : Le Département de Biologie Animale doit remettre à la Direction du Système des Aires Protégées, en quatre (04) exemplaires EN FRANÇAIS, le rapport préliminaire à la fin de sa mission et le rapport final avec les résultats des recherches au plus tard deux ans après la mission.
Le bénéficiaire de la présente autorisation doit :

- faire viser la présente par la Direction Régionale de l'Environnement et des Forêts Ihorombe et/ou CEF Ihosy, Triage Ranohira avant toute descente sur terrain, conformement à la note **n° 394- 10/MEF/SG/DGF/DVRN/SGFF du 18 Mai 2010.**
- prendre le ticket d'entrée auprès de MNP (Madagascar National Parks) dans le cas où la recherche s'effectue dans les Aires Protégées gérées par celui-ci.

Antananarivo, le 02 DEC 2011

AMPLIATIONS :

- CAFF/CORE
- DCAI
- DREF : Ihorombe
- CEF Ranohira
- Triage Ranohira
- Communes concernées
  « Pour contrôle et suivi »

- DBA
  « Pour le rapport »

LE DIRECTEUR DE LA CONSERVATION DE LA BIODIVERSITE ET DU SYSTEME DES AIRES PROTEGEES

MINISTERE DE L'ENVIRONNEMENT, DES FORETS ET DU TOURISME – DIRECTION GENERALE DE L'ENVIRONNEMENT ET DES FORETS – DIRECTION DU SYSTEME D'AIRES PROTEGEES

ANDRIANARIVELO-RAZAFY Mamy

REPOBLIKAN'I MADAGASIKARA
Fitiavana-Tanindrazana -Fandrosoana

MINISTERE DE L'ENVIRONNEMENT ET DES FORETS
B.P: 3948, Antsahavola – ANTANANARIVO – 101-
Tel: (261 20) 22 411 55 – Fax: (261 20) 22 419 19

AUTORISATION DE :

x - RECHERCHE
- ETUDE

N° 032 /12/MEF/SG/DGF/DCB.SAP/ SCBSE
NOM RAMASINDRAZANA
PRENOMS Beza
ADRESSE B.P 906 Antananarivo
FONCTION Chercheur
ACCOMPAGNE DE : Steve Goodman, un représentant du CAFF/CORE.

ORGANISME TUTELLE : Département de Biologie Animale (DBA)

EST AUTORISE(E) A FAIRE DES RECHERCHES / ETUDES DANS :
La grotte de Fandanana (Fandriana), Itampolo, Sarodrana, Androka Ankililaoka en dehors des aires protégées (SAPM)
« L'accès dans les forêts transferées à un comité de géstion fera l'objet de négociation avec ce dernier ».

MENTION SPECIALE EVENTUELLE:
Etude des hémoparasites chez les chauves-souris Malagasy.
Capture avec relâche d'aun maximum 10 specimens par especes de chauves-souris après études morphologique, morphométrique, parasitologique et prélèvement d'échantillons de sang et tissu pour étude moléculaire.
Collecte de deux individus par espèce des spécimens difficile à identifier.
Collecte d'ectoparasites.
Echantillonnage des insectes nocturnes (diptères).

DUREE : Six (06) mois.

N.B Le Département de Biologie Animale doit remettre à la Direction du Système des Aires Protégées, en quatre (04) exemplaires EN FRANÇAIS, le rapport préliminaire à la fin de sa mission et le rapport final avec les résultats des recherches au plus tard deux ans après la mission.
Le bénéficiaire de la présente autorisation doit :
- faire viser la présente par la Direction Régionale de l'Environnement et des Forêts Atsimo Andrefana, Haute Matsiatra et/ou CEF concernée avant toute descente sur terrain, conformement à la note **n° 394- 10/MEF/SG/DGF/DVRN/SGFF du 18 Mai 2010.**
- prendre le ticket d'entrée auprès de MNP (Madagascar National Parks) dans le cas où la recherche s'effectue dans les Aires Protégées gérées par celui-ci.

Antananarivo, le 02 FEV 2012

AMPLIATIONS :
- CAFF/CORE
- DCAI
- DREFs: Aand, Hm
- CEF : concernées
- Communes concernées

« Pour contrôle et suivi »

- DBA
« Pour le rapport »
Vu au passage à Toliara

[illegible]AZAKA Victor M. R.
Ingénieur des Eaux et Forêts

LE DIRECTEUR DE LA CONSERVATION DE LA BIODIVERSITE ET DU SYSTEME DES AIRES PROTEGEES

[illegible]NY Laurette [illegible]
Ingénieur des Eaux et Forêts

REPOBLIKAN'I MADAGASIKARA
Fitiavana-Tanindrazana–Fandrosoana

SECRETARIAT GENERAL

DIRECTION GENERALE DES FORETS

DIRECTION DE LA CONSERVATION DE LA BIODIVERSITE ET DU SYSTEME DES AIRES PROTEGEES

AUTORISATION DE :
x- RECHERCHE
- ETUDE

N° 067 /12/MEF/SG/DGF/DCB.SAP/ SCBSE
NOM RAMASINDRAZANA
PRENOMS Beza
ADRESSE B.P 906 Antananarivo
FONCTION Chercheur
ACCOMPAGNE DE : Steve Goodman, un représentant du CAFF/CORE.

ORGANISME TUTELLE : Département de Biologie Animale (DBA)

EST AUTORISE(E) A FAIRE DES RECHERCHES / ETUDES DANS :
La grotte d'Anjohibe, la forêt de Menabe Central en dehors CFPF et en dehors des Aires Protégées (SAPM)
« L'accès dans les forêts transférées à un comité de gestion fera l'objet de négociation avec ce dernier ».

MENTION SPECIALE EVENTUELLE:
Etude des hémoparasites chez les chauves-souris de la partie occidentale Malagasy.
Capture avec relâche d'au maximum 10 specimens par espèce de chauves-souris après études morphologique, morphométrique, parasitologique et prélèvement d'échantillons de sang et tissu pour étude moléculaire
Collecte de deux individus par espèce des spécimens difficile à identifier
Collecte d'échantillons des insectes nocturnes (diptères) nourriture des chauves-souris.

DUREE : Six (06) mois.

N.B Le Département de Biologie Animale doit remettre à la Direction du Système des Aires Protégées, en quatre (04) exemplaires EN FRANÇAIS, le rapport préliminaire à la fin de sa mission et le rapport final avec les résultats des recherches au plus tard deux ans après la mission.
Le bénéficiaire de la présente autorisation doit :
- faire viser la présente par la Direction Régionale de l'Environnement et des Forêts Boeny, Menabe, et/ou CEF concernée avant toute descente sur terrain, conformement à la note **n° 394- 10/MEF/SG/DGF/DVRN/SGFF du 18 Mai 2010.**
- prendre le ticket d'entrée auprès de MNP (Madagascar National Parks) dans le cas où la recherche s'effectue dans les Aires Protégées gérées par celui-ci.

Antananarivo, le 06 MARS 2012

AMPLIATIONS :
- CAFF/CORE
- DCAI
- DREFs: Boe, Mnb
- CEF : concernées
- Communes concernées

« Pour contrôle et suivi »

- DBA

« Pour le rapport »

LE DIRECTEUR DE LA CONSERVATION DE LA BIODIVERSITE ET DU SYSTEME DES AIRES PROTEGEES

MINISTERE DE L'ENVIRONNEMENT ET DES FORETS — DIRECTION GENERALE DE L'ENVIRONNEMENT ... DU SYSTEME DES AIRES PROTEGEES

RASOAVAHINY Laurent Hermine
Ingénieur des Eaux et Forêts

MINISTERE DE L'ENVIRONNEMENT ET DES FORETS

REPOBLIKAN'I MADAGASIKARA
Fitiavana–Tanindrazana–Fandrosoana

SECRETARIAT GENERAL

DIRECTION GENERALE DES FORETS

DIRECTION DE LA CONSERVATION DE LA BIODIVERSITE ET DU SYSTEME DES AIRES PROTEGEES

AUTORISATION DE :
x- RECHERCHE
- ETUDE

N° 194 /12/MEF/SG/DGF/DCB.SAP/ SCB

NOM RAMASINDRAZANA
PRENOMS Beza
ADRESSE B.P 906 Antananarivo
FONCTION Chercheur
ACCOMPAGNE DE : Steve Goodman, un représentant du CAFF/CORE.

ORGANISME TUTELLE : Département de Biologie Animale (DBA)

EST AUTORISE(E) A FAIRE DES RECHERCHES / ETUDES DANS :
Le reseau des Parcs Nationaux : Ankarana, Bemaraha, Namoroka, Tsimanampetsotsa, Ambohitantely.

MENTION SPECIALE EVENTUELLE:
Etude des hématoparasites chez les chauves-souris Malgaches.
Capture avec relâche d'au maximum 10 specimens par espèce de chauves-souris après études morphologique, morphométrique, parasitologique et prélèvement d'échantillons de sang et tissu pour analyses moléculaire.
Collecte d'ectoparasites.
Collecte de deux individus par espèce de chauves-souris pour les spécimens difficile à identifier.
Collecte d'échantillons des insectes nocturnes (*diptères*), nourriture des chauves-souris, pour dépistage des parasites.

DUREE : Six (06) mois.

N.B Le Département de Biologie Animale doit remettre à la Direction du Système des Aires Protégées, en quatre (04) exemplaires EN FRANÇAIS, le rapport préliminaire à la fin de sa mission et le rapport final avec les résultats des recherches au plus tard deux ans après la mission. Rapports en versions papier et électronique.
Le bénéficiaire de la présente autorisation doit :
- faire viser la présente par la Direction Régionale de l'Environnement et des Forêts Diana, Melaky, Atsimo Andrefana, Analamanga
  et/ou CEF concernées avant toute descente sur terrain,
  conformement à la note **n° 394- 10/MEF/SG/DGF/DVRN/SGFF du 18 Mai 2010.**
- prendre le ticket d'entrée auprès de MNP (Madagascar National Parks) dans le cas où la recherche s'effectue dans les Aires Protégées gérées par celui-ci.

Antananarivo, le 13 AOUT 2012

AMPLIATIONS :
- CAFF/CORE
- DCAI
- DREFs : Diana, Mlk, Aand, Anlg
- CEF : concernées
- MNP
- AP concernées
- Communes concernées
« Pour contrôle et suivi »

- DBA
« Pour le rapport »

LE DIRECTEUR DE LA CONSERVATION DE LA BIODIVERSITE ET DU SYSTEME DES AIRES PROTEGEES

RASOAVAHINY Lourette Hermine
Ingénieur des Eaux et Forêts

Annexe 10 – Demande de Service CNDRS

CENTRE NATIONAL DE DOCUMENTATION ET DE RECHERCHE SCIENTIFIQUE

Musée National des Comores

Archives nationales
Bibliothèque nationale

Sciences humaines
Histoire naturelle

Réf. CNDRS 021/10

Moroni, le 1er mars 2010

Vu et accordé
DNST

A
Monsieur le Directeur de la Sureté Nationale
S/C
Monsieur le Secrétaire Général du Ministère de l'Education Nationale et de la Recherche.

VISA

Objet : Demande de service

Monsieur le Directeur,

J'ai l'honneur de vous informer de l'arrivée aux Comores du 5 au 13 mars 2010 d'une équipe scientifique pluri-disciplinaires(épidémiologiste, virologiste et entomologiste médicale), du laboratoire du Centre de Recherche et de Veille sur les Maladies Emergentes (CRVOI) basé à l'île de la Réunion pour effectuer des études sur des maladies émergeantes probablement transmises à partir des espèces animales hôtes comme les chauves-souris.

Cette équipe sera constituée comme suit :

GUERNIER Vanina
LAGADEC Erwan
GOODMAN Steven
RAMASINDRAZANA Beza

Pour ce faire, je vous prie de bien vouloir leur accorder un visa couvrant la période de leur séjour aux Comores

Vous souhaitant bonne réception, je vous prie, d'agréer, Monsieur le Directeur, l'expression de mes cordiales salutations.

Avis favorable

Directeur Général du CNDRS
S/C
Le Directeur par intérim
Mohamed Assoumane

CNDRS, B.P. 169, MORONI, UNION DES COMORES
Tél. : (269) 773 81 53 Fax : (269) 773 92 00 E-mail : cndrs@comorestelecom.km

Annexe 4 – Autorisation d'exportation

UNION DES COMORES

Unité – Solidarité- Développement

MINISTERE DE L'EDUCATION NATIONALE DE L'ENSEIGNEMENT SUPERIEUR ET DE LA RECHERCHE

UNIVERSITE DES COMORES

Faculté des Sciences ET Techniques

AUTORISATION

Dans le cadre du programme Faune Sauvage-Océan Indien porté par le Centre de Recherche et de Veille sur les maladies émergentes dans l'Océan Indien (CRVOI), la Faculté des Sciences et Techniques de l'Université des Comores, représentée par son Doyen le Docteur Ahmed Ouledi, expédie au CRVOI quatre vingt dix (90) spécimens de mammifères pour des identifications et des analyses scientifiques (voir annexe).

Ces spécimens seront apportés par le Docteur LAGADEC Erwan.

Ces échantillons ne figurent pas sur la liste du CITES.

Etablie pour servir et valoir ce que de droit.

LE DOYEN DE LA FST.

Dr AHMED Ouledi

Spécimens pour exportation collectés dans le contexte du programme Faune Sauvage-Océan Indien en mars 2010.

NON CITES

Destination : Centre de Recherche et de Veille sur les maladies émergentes dans l'Océan Indien
Technopole
2 rue Maxime Rivière
97490 Sainte Clotilde
La Réunion

Espèce	Nombre de spécimens (avec tissus, sérum et ectoparasites)
Roussettus obliviosus	30
Miniopterus sp.	20
Chaerephon sp.	40
**Nombre total de spécimens**	**90**
